# Variance and invariance of neuronal long-term representations

**DOI:** 10.1098/rstb.2016.0161

**Published:** 2017-03-05

**Authors:** Claudia Clopath, Tobias Bonhoeffer, Mark Hübener, Tobias Rose

**Affiliations:** 1 Bioengineering Department, Imperial College London, South Kensington Campus, London SW7 2AZ, UK; 2 Max Planck Institute of Neurobiology, Am Klopferspitz 18, 82152 Martinsried, Germany

**Keywords:** plasticity, stability, chronic electrophysiology, Ca^2+^ imaging, two-photon imaging, circuit model

## Abstract

The brain extracts behaviourally relevant sensory input to produce appropriate motor output. On the one hand, our constantly changing environment requires this transformation to be plastic. On the other hand, plasticity is thought to be balanced by mechanisms ensuring constancy of neuronal representations in order to achieve stable behavioural performance. Yet, prominent changes in synaptic strength and connectivity also occur during normal sensory experience, indicating a certain degree of constitutive plasticity. This raises the question of how stable neuronal representations are on the population level and also on the single neuron level. Here, we review recent data from longitudinal electrophysiological and optical recordings of single-cell activity that assess the long-term stability of neuronal stimulus selectivities under conditions of constant sensory experience, during learning, and after reversible modification of sensory input. The emerging picture is that neuronal representations are stabilized by behavioural relevance and that the degree of long-term tuning stability and perturbation resistance directly relates to the functional role of the respective neurons, cell types and circuits. Using a ‘toy’ model, we show that stable baseline representations and precise recovery from perturbations in visual cortex could arise from a ‘backbone’ of strong recurrent connectivity between similarly tuned cells together with a small number of ‘anchor’ neurons exempt from plastic changes.

This article is part of the themed issue ‘Integrating Hebbian and homeostatic plasticity’.

## 1. Introduction

The building blocks of the brain are in constant flux at the subcellular, cellular and circuit level. Synaptic and non-synaptic proteins are mobile [1] and rapidly turn over on the scale of hours to days [2]. Individual synapses continuously change their size and strength both *in vitro* and *in vivo* [3–5]. Most notably, however, the mature brain appears to continuously rewire itself, even without experimental intervention [6,7]. This is evident from the perpetual turnover of dendritic spines, small protrusions from the parent dendrites of most cortical neurons that are commonly used as proxies for excitatory synapses. Depending on the cell types and brain regions investigated, dendritic spines are gained and lost at rates ranging from approximately 1% per day in primary visual cortex [8] over approximately 5% per day in the CA1 region of hippocampus [9] to up to approximately 15% per day in primary somatosensory cortex [10] (but see [6,11,12] for potential pitfalls of these quantifications). How, then, is the brain able to maintain stable computational capabilities, stable representations of external and internal features and stable behavioural performance when facing such variability?

**Figure 1.**
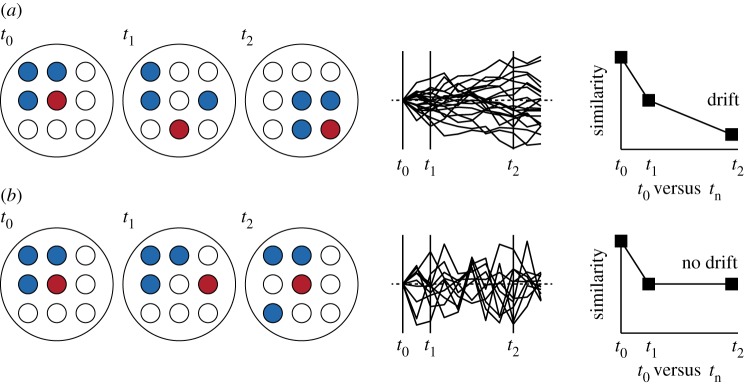
Variability and drift of neuronal representations. (*a*) Example of single-cell drift. Cartoon showing a hypothetical population of nine cells at three different time points (*t*_0_, *t*_1_, *t*_2_). Cells are ‘tuned’ to three different features (blue, red and white). The average population output remains stable over time (3× blue, 1× red, 5× white), but the tuning of individual cells becomes progressively dissimilar (right panel) to the first time point (illustrated as random walk in the middle panel). (*b*) Example of variability without drift. As in (*a*), population output remains stable and cells are variable. However, single-cell tuning remains stable in the long term and does not become progressively dissimilar (right panel) with respect to the first time point (illustrated as random walk with mean reversion in the middle panel). Note that the differences between time points *t*_0_ and *t*_1_ are indistinguishable in (*a*) and (*b*), showing that recordings at only two time points are insufficient to distinguish between drifting and non-drifting representations.

It has long been appreciated that stability in dynamic biological systems, like the brain, can arise from unstable constituents (as reviewed in [13]). Higher-order stability is observed, for instance, on the level of synaptic population strength [5] and on the level of long-term stable representations of stimulus features by neuronal populations [14,15]. Especially in the latter case, however, it is debated whether stable population coding results from invariant stimulus selectivities of individual neurons or from noisy and potentially drifting single-cell responses that are ‘averaged out’ over a large number of neurons, causing overall robust circuit performance [16] (figure 1).

In order to distinguish between these alternatives, the activity of the same individual cells and cell populations has to be followed over multiple time points (figure 1) [16]. The technology necessary to record action potential (AP) firing longitudinally has improved vastly in recent years. Chronic electrophysiological recordings have so far suffered from electrode drift and gliosis, which rendered it difficult to unambiguously associate the constantly changing electrical signals with the same neuron. However, by establishing robust electrode-mounting procedures and stringent criteria for isolating and following individual units over time, several groups have acquired longitudinal spiking data from a small number of neurons in head-fixed or freely moving animals by intermittent recordings over days and even years [17–21]. More recently, further refined techniques have emerged that take advantage of state-of-the-art data processing methods to solve the problem of longitudinal unit identification by gap-free long-term recordings that were analysed by automated spike sorting algorithms [22,23]. Dhawale *et al.* were able to automatically follow a large number of individual neurons for an average of approximately 4 days with a small fraction of cells that could be followed over extended time periods (e.g. approx. 5 out of 1000 for up to a month [22]). Current efforts to increase the density of electrode recording sites [24] are likely to yield higher numbers of chronically recorded cells in the future. To further overcome electrode drift, injectable mesh electrodes have been developed that minimize shear forces relative to the surrounding tissue [25]. Using this approach, exceptionally stable long-term single-cell recordings (longer than eight months) have been demonstrated recently [26]. This promising technique should further minimize variability of technical origin in the assessment of representational stability in the brain.

Functional imaging provides an increasingly popular alternative to chronic electrophysiology in cases where high temporal recording fidelity is not needed. Imaging allows the unambiguous identification of hundreds [14,15,27,28] or even thousands [29] of the same neurons over essentially arbitrary time-intervals. First and foremost, two-photon microscopy [30] of genetically encoded Ca^2+^ indicators (GeCIs) now permits the recording of activity-evoked neuronal Ca^2+^ influx, in some cases approaching single AP resolution [31–34]. After the first proof-of-principle demonstration of chronic functional two-photon imaging in mouse visual cortex [35], the technique has now been used widely in various movement-restricted animal models over repeated sessions during normal experience and after experimental intervention [14,15,18,27,29,31,36–49]. Importantly, cell-type specific labelling made it possible to follow cells with specific genetic makeup (e.g. excitatory or inhibitory neurons [37,41,42]) or specific projection patterns (e.g. neurons projecting to or from specific cortical areas [38,39]). However, two-photon imaging of freely moving animals, a standard electrophysiological approach, has not been widely adopted owing to the technically challenging miniaturization necessary to achieve head-mounting on small laboratory animals [50,51]. More recently, small and simple wide-field fluorescence microscopes have been developed that allow longitudinal fluorescence imaging of a large number of identified neurons in freely moving animals [28,52–55], albeit sacrificing the optical sectioning and deep tissue penetration capabilities of two-photon microscopy [56].

Electrophysiological and imaging techniques have their own unique advantages and disadvantages for long-term recordings with regard to the reliability of cell-matching across sessions, signal-to-noise ratio, temporal fidelity, cell selection biases, response linearity, signal extraction complications, long-term cell health, tissue damage, etc. [16,22,34,57]. All these factors are likely to directly affect the assessment of neuronal variability. Furthermore, longitudinal recordings of cellular activity have been performed using a plethora of different experimental paradigms in multiple preparations—from *in vitro* recordings in dissociated cultures [58,59] to *in vivo* recordings in primary and higher sensory areas [14,15,18,19,29,35,36,38,39,41–44,46,47,49,60], motor areas [22,27,37,48,55,61–65], striatum [22] and hippocampus [28,40,45,52,66–69]. Comparing the variability of neuronal feature selectively therefore is challenging—nevertheless, we would first like to provide an update on long-term neuronal variability assessments (for a previous in-depth review see [16]; for a review on short-term trial-to-trial variabiliy see [70]).

## 2. Stability versus variability in motor cortex

The question of whether stable behavioural performance results from stable single-cell representations or from stability only on the population level is of particular relevance in primary motor cortex (M1). Here, it has direct implications for the design and prospective long-term performance of brain–machine interfaces (BMIs) for the control of motor prosthetics [61,65,71]. The stability of movement representations by individual M1 neurons has therefore been addressed comparatively well, yielding, however, conflicting results. Neuronal selectivity for directional reaching movements has been studied under baseline conditions and in a task requiring reversible sensorimotor adaptation in macaque monkeys [61,62,72]. Although motor performance continuously increased during task execution and became highly stereotypic, directional movement tuning of individual neurons was found to be variable during baseline but also after learning [61,62]. This led to the conclusion that even stereotypical movements are controlled using a population code with redundant and drifting neuronal constituents, rendering the specific tuning of individual neurons largely irrelevant [62].

Other groups obtained lower estimates of variability across individual trials and across recording sessions using a similar experimental paradigm [63–65]. Especially, the strong dependence of directional tuning curves on measurement noise makes it difficult to judge the actual variability of representations in M1 [63]. Very recent electrophysiological evidence appears to rather support highly stable single-cell tuning features in M1 and dorsolateral striatum for trained movements [22]. Continuous recordings over weeks and months showed that neuronal activity and motor representations of a learned lever-pull task were highly reproducible and essentially drift-free [22].

By contrast, a chronic two-photon Ca^2+^ imaging study assessing sensorimotor representations in mouse M1 during a whisker-based object detection task [27] rather supports unstable single-cell tuning. Most neurons, again, did not consistently represent a single behavioural feature (licking, whisking and touch) during repeated imaging. Furthermore, a decoding algorithm trained on AP responses from an early session performed progressively worse when applied to later time points, indicating single-cell drift (cf. figure 1*a*). Similar results were obtained in later imaging studies, where most excitatory neurons did not consistently represent movement-related activity across sessions [37,48]. Interestingly, the activity of inhibitory neurons was significantly less variable [37]. Recent Ca^2+^ imaging data obtained with miniature wide-field microscopes provide further support for a stable population motor code with unstable single-cell participation [55]. In HVC, a pre-motor area that drives stable learned song in zebra finches, the firing patterns of individual neurons were highly variable whereas average population activity was stable. Again, inhibitory neurons showed less variability than excitatory neurons [55].

Similar to previous electrophysiological studies [64,73,74], neuronal activity in many Ca^2+^ imaging studies became more invariantly task-related with training [27,37,48], with deep layer 5a (L5a) neurons showing more improvement than supragranular L2/3 [48]. Furthermore, the ensemble behaviour–response association during learning of a task stabilized, which led to higher across-session correlations in movement-related single-cell activity with increasing task performance [27,37]. Some of the discrepancies in reported single-cell stability may therefore be owing to different levels of task proficiency (also see [71]) or owing to cell-type biases as a result of different cortical recording depths [16].

## 3. Stability versus variability in hippocampus

The hippocampal representation of space over time provides further evidence that stability and variability of neurons and of neuronal populations are actively regulated and of potentially high functional and behavioural relevance. Also in this structure, longitudinal studies have first provided seemingly contradictory evidence.

**Figure 2.**
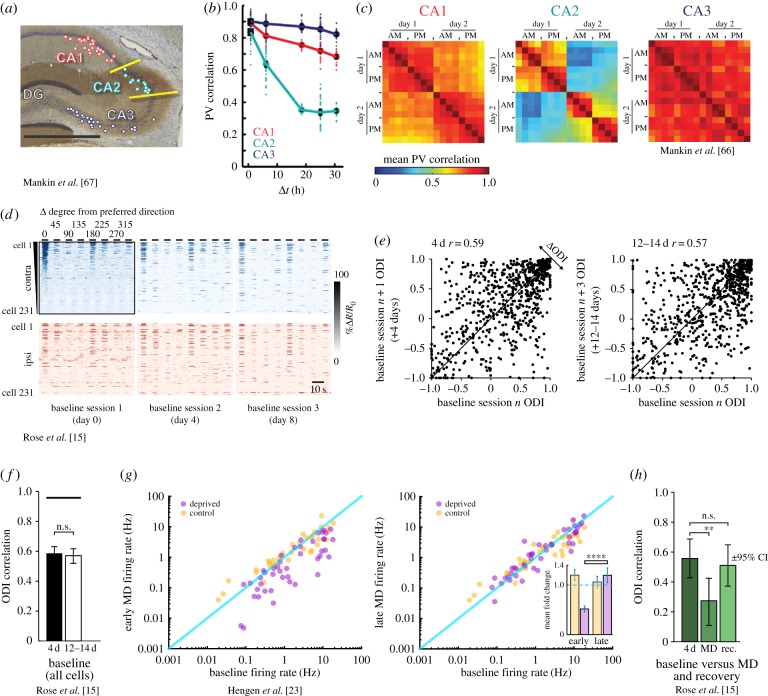
Variance and invariance of neuronal representations in hippocampus and visual cortex. (*a*) Chronic electrophysiology in rat hippocampus *in vivo* [67]. Example brain section showing the CA1, CA2 and CA3 regions and the positions (coloured dots) of extracellular recording electrodes from Mankin *et al.* [67]. (*b*) Correlation of the activity of all longitudinally recorded cells (population vector, PV) over different time-intervals for CA1 (red), CA2 (cyan) and CA3 (blue). Activity was recorded while rats were foraging for food in a familiar environment. Although the CA3 PV correlation does not drift, CA2 shows a progressively dissimilar PV with increasing time-intervals and therefore shows prominent drift. (*c*) Pairwise PV correlation matrices over all measurement time points in the same environment. The shift towards ‘colder’ colours indicates the gradual PV decorrelation over time in CA1 and, more pronounced, CA2. (*d*) Chronic two-photon Ca^2+^ imaging in mouse binocular V1 [15]. Shown are eye-specific fluorescence ratio changes in response to moving grating stimulation (eight directions) in 231 excitatory L2/3 neurons (one animal) over three baseline sessions (8 days). All responses are sorted for preferred direction (horizontally) and contralateral eye (blue) response magnitude (vertically) in the first session. Although overall amplitude and preferred orientation ranks are maintained over sessions and are matched between eyes, some degree of tuning variability across sessions and eyes is visible. (*e*) Scatter plots showing the correlation of eye-specific tuning (ocular dominance index, ODI) of the same individual neurons between sessions spaced either 4 days or 12–14 days apart. (*f*) Quantification of data in (*e*). Although variability between sessions is larger than expected from within-session variability (black line), the ODI correlation does not progressively decline. Eye-specific tuning therefore does not show drift over two weeks during normal sensory experience. (*g*) Neurons in monocular V1 of juvenile rats maintain a cell-specific homeostatic set point after monocular deprivation (MD) [23]. Continuous electrophysiological recordings show that the firing rate of the same individual neurons drops early after MD relative to the pre-MD baseline rate (left scatter plot, purple symbols). The firing rate of the same cells recovers to baseline during late MD (right scatter plot). Inset: quantification of the data. (*h*) Individual neurons in adult mice recover their initial eye-specific tuning after MD [15]. Correlation of neurons that underwent a significant ODI shift after MD during baseline (sessions 4 days apart), baseline and MD, and baseline and recovery. Adapted, with permission, from [67] (*a–c*), [15] (*d*, *e*, *f* and *h*), and [23] (*g*).

Early chronic electrophysiological recordings from a small number of neurons in the hippocampal CA1 region in freely moving rats showed that the spatial firing fields of individual place-cells are highly stable over weeks [75]. By contrast, later recordings in mouse CA1 showed less stable place preference [69,76], suggesting potential species-dependent differences. However, similar to the task-dependent stabilization in motor cortex, chronically recorded rate maps from individual CA1 place-cells were far better correlated across sessions when animals were actively engaged in a visuospatial place-preference task than when animals had no reason to pay attention to spatial cues [69,76]. Importantly, place field stability in rats has been shown to be differentially regulated across hippocampal regions. Although CA2 population activity exhibited prominent drift over multiple recording sessions, spatial preference of cells in CA3 was largely maintained, while CA1 showed intermediate stability [66,67] (figure 2*a–c*). These and similar data [77] led to the proposal that the gradual decorrelation (i.e. drift, cf. figure 1*a*) of hippocampal ensemble activity over hours and days provides a ‘timestamp’ for spatial information, allowing, for instance, the encoding of the temporal order of event sequences [66,67,77].

The recent development of miniaturized fluorescence microscopes [54] allowed long-term recordings from hundreds of hippocampal CA1 neurons in freely moving mice over months [28,52]. Using different GeCI iterations of the GCaMP family, these studies strongly supported the notion of a temporally evolving representation of space in hippocampal CA1. Of all cells, approximately 20% had significant place fields. Of these, only approximately 25% still expressed place preference after 5 days, which further declined to approximately 15% thirty days later [28]. The fraction of cells that maintained spatial tuning, however, also maintained their original place preference [28,52], indicating a certain degree of stability in otherwise drifting ensembles. A recent two-photon imaging study of head-fixed mice running on a conveyor belt with spatial multisensory cues provided an even more fine-grained picture of region-dependent variability in the hippocampus [40]. Superficial CA1 neurons showed significantly less variability of spatial tuning over time than deeper CA1 neurons [40]. Furthermore, granule cells of the dentate gyrus were also shown to express drifting behavioural state preference (i.e. running versus not running) [45] over days.

## 4. Stability versus variability in sensory cortex

It can be argued that variability in the motor cortex may be important for motor learning [62], and drift in the hippocampus may provide timestamps of spatially relevant events [66,67,77]. However, it stands to reason that primary sensory areas should faithfully encode lower-order statistics of the external world to provide downstream areas with stable access to sensory information. Until recently, however, very few studies attempted chronic recordings in primary sensory areas (e.g. [17,18]). Only recent years have seen a prominent increase in the number of mostly imaging studies addressing the stability and plasticity of neuronal representations in the visual system [15,19,35,36,38,41,43,44,46], barrel cortex [14,29,39,47,60] and auditory cortex [42].

The tuning curves of neurons in primary somatosensory cortex (S1) were found to be surprisingly variable in an initial two-photon Ca^2+^ imaging study in anesthetized mice [14]. Although the average spike-rate of the population was constant, and the overall activity level of individual L2/3 cells was similar over days, the preferred responsiveness to stimulation of either one of two neighbouring whiskers was only weakly correlated over sessions spaced days apart [14]. Later studies, however, showed less variable evoked activity in slightly different paradigms. Using single-cell electrophysiological recordings, preference for ipsi- or contralateral whisker stimulation was shown to be stable over 14 days in the small number of cells tested [60]. While confirming a certain degree of session-to-session variability, later Ca^2+^ imaging data also showed that some representations in S1 are largely drift-free because the performance of a population decoder of whisker deflection frequency did not progressively decline when trained on an early session and applied to later sessions [47].

A recent imaging study recorded chronically from approximately 75% of all supragranular neurons of a single barrel in mouse S1 (approx. 12 000 cells per mouse) during learning of a whisker-based object localization task [29]. Similar to motor cortex [27], the correlation between sensorimotor variables (whisking and touch) and neural activity was weaker and more variable during initial training sessions than during later sessions when animals reached task proficiency [29]. Similar results were obtained in a comparable study by Chen *et al.* [39], but they found additionally that neurons show differential variability based on their projection pattern. Retrogradely labelled L2/3 neurons projecting from S1 to M1 showed a significantly higher reduction of variability with increasing task proficiency than neurons projecting to secondary somatosensory cortex (S2) [39].

Variability of visual tuning in primary visual cortex (V1) has rarely been addressed. Chronic extracellular recordings in macaque monkeys showed that the similarity of preferred orientation (and direction) of moving grating stimulation across days was significantly higher for isolated units putatively belonging to the same cell in comparison with units that were most likely representing different neurons [18]. Very recently, we showed in a chronic two-photon imaging study that the session-to-session variability of visual tuning properties (ocular dominance (OD), preferred orientation, and orientation selectivity) of excitatory L2/3 neurons in anesthetized and awake mice was larger than expected from within-session trial-to-trial variability [15] (figure 2*d–f*). Importantly, however, the correlation of tuning properties did not progressively decline over time. The same cells were equally well correlated over a shorter intersession-interval (4 days) as over a far longer interval (12–14 days) [15] (figure 2*e,f*). In contrast with motor cortex (but see [27]) and hippocampus [28,52,66,67], but comparable to barrel cortex [47], excitatory L2/3 neurons in visual cortex therefore did not show overt drift during normal sensory experience (cf. figure 1).

Chronic imaging of activity in L2/3 of V1 during a visual discrimination task [36] yielded results comparable to learning in M1 [27,37] and S1 [29,39]. Although the fluctuations in session-to-session selectivity to two task-relevant visual stimuli were large during initial training sessions (only approx. 20–50% of cells maintained selectivity across sessions), significantly more cells became invariantly discriminative with increasing task proficiency [36].

Further evidence supporting the stabilization of behaviourally salient sensory representations comes from long-term electrophysiological recordings in the temporal lobe of macaque monkeys [19]. Here, individual cells preferably respond to the visual feature combinations of individual faces, which are of high behavioural relevance for social animals as macaques [78]. The data of McMahon *et al.* [19] suggest that even though selectivity for individual faces is learned [78], face representation of individual neurons appears to be surprisingly stable over months.

## 5. Perturbation resistance of visual cortex

The drift-free representation of sensory features in mature V1 [15] and S1 [47] either suggests a high degree of resistance to constitutive plasticity during normal sensory experience, or that plasticity is effectively suppressed [79–81]. What if the system is taxed with a strong but reversible perturbation that is known to induce prominent experience-dependent rewiring even in mature circuits [81,82]? Two recent studies addressed this question in rodent V1 using monocular deprivation (MD), a well-established paradigm of experience-dependent plasticity [15,23,79–81].

Hengen *et al.* [23] performed continuous electrophysiological recordings from L2/3 cells in monocular V1 (mV1) of juvenile rats before and after closing the eye providing input to this region. MD is known to lead to an immediate drop in visual drive from the deprived eye, which results in a pronounced initial decrease in the average firing rate in mV1 [23,83]. Shortly after this drop in activity, however, the average population activity returns to its pre-MD level [83]. This is commonly interpreted as a result of both cell-autonomous and network level homeostatic processes that work together to keep cellular firing within a specific working regime ([23,83]; also see review of [84,85]). Hengen *et al.* [23] show now that individual neurons did not only maintain stable average firing rates before MD, but that these cells also faithfully returned to their initial firing rate after the transient drop in activity (figure 2*g*). The homeostatic set point is therefore both cell specific and highly perturbation resistant (see [85]). Interestingly, a precise cellular homeostatic set point seems to be a feature of intact, mature circuits, because this finding is in contrast with recent data from dissociated cell cultures. Here, individual neurons did not return precisely to their initial activity level after homeostatic recovery from depressed activity, even though the population average firing rate recovered [58,59].

MD induces a prominent shift towards the non-deprived eye in binocular V1 (bV1) of adult mice. This shift is accompanied by functional and structural circuit rearrangements [8,80,81]. On the population level, this shift is known to be fully reversible after a period of binocular vision following MD [8,80,81,86]. However, MD-induced circuit plasticity is a strong perturbation that may be expected to lead to an irreversible restructuring of circuits in V1 and a dynamic rearrangement of individual cellular tuning features after recovery. This would probably render the post-recovery tuning of individual cells very different from their pre-MD state. However, using long-term two-photon Ca^2+^ imaging, we showed that the opposite is the case [15]. Even though neurons showed a prominent shift towards the deprived eye after MD, their individual OD precisely returned to the pre-MD state within the fluctuations expected from baseline variability [15] (figure 2*h*).

## 6. Perturbation resistance of visual cortex—a circuit model

The latter three findings—(i) drift-free sensory representation during normal experience [15], (ii) long-term maintenance of a cell-specific homeostatic set point [23] and (iii) precise recovery of initial tuning features after prominent experience-dependent plasticity [15]—show that the mature visual cortex achieves a high degree of robustness to both constitutive and experience-dependent plasticity. How could such robustness arise?

It has long been known that specific network structures can lead to stable cellular activity patterns that are robust to noise (as reviewed in [13]). We therefore explored whether the functional connectivity of V1 itself may convey stability and perturbation resistance to neuronal representations. Electrophysiological assessments of connectivity have shown that the overall probability of excitatory cell-to-cell connectivity is low, with a few strong and often reciprocal connections between subsets of neurons [87–90]. Cellular interconnectivity is strongly correlated with functional similarity, and cells with similar stimulus preferences form interconnected subnetworks [88–90].

**Figure 3.**
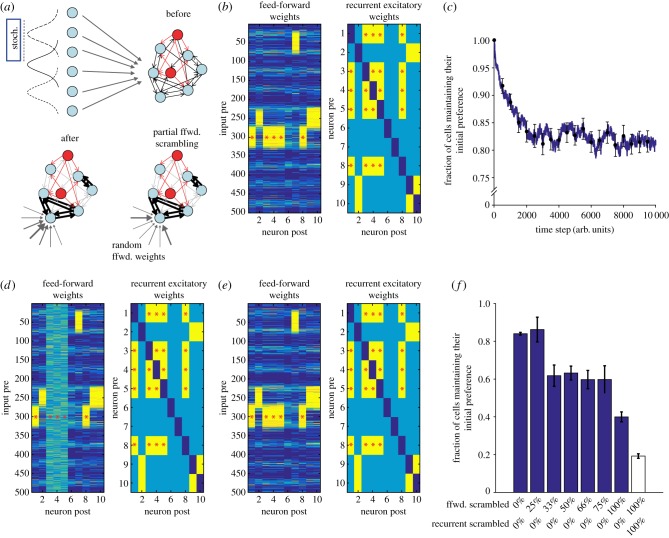
Model of a plastic microcircuit. (*a*) A densely interconnected network of 10 excitatory (light blue) and three inhibitory neurons (red) receives feed-forward (ffwd) inputs from 500 Poisson spike trains with a Gaussian profile of firing rates. The centre of the Gaussian was shifted randomly to one out of 10 possible locations in stimulus space every model iteration (schematic network before (right) and after 10 000 model iterations (bottom left); see detailed methods and parameters in Clopath *et al.* [91]). (*b*) Network structure after 10 000 iterations of the model. Mean feed-forward weights (left) and recurrent excitatory weights (right) averaged over 1000 steps. The clustering of feed-forward weights (left) indicates that the neurons developed individual stimulus preferences. These ‘receptive fields' were expressed as strong synaptic weights of the feed-forward inputs corresponding to one of the 10 stimulus positions (synaptic weights from weak, blue, to strong, yellow). The recurrent weights (right) were classified as weak (less than two-thirds of the maximal weight, light blue) or strong (more than two-thirds of the maximal weight, yellow). The diagonal is dark blue, as autaptic connections are not allowed in the model. Red asterisks indicate a recurrently connected example ensemble with similar receptive fields (cells 1, 3, 4, 5 and 8). (*c*) Baseline stability of the model. After the first 10 000 iterations of the model, it was allowed to continuously evolve over 10 000 further time steps. The similarity to the state after the first 10 000 steps decreased initially, but then plateaued. The model therefore did not show overt drift (cf. figure 1*b*). (*d*) As a perturbation, the feed-forward connections to three (cells 3, 4, 5) out of the five cells in the co-tuned example ensemble in (*b*)(red asterisks) were set to random values (see (*a*), bottom right). (*e*) The network converged back from the perturbation (10 000 further time steps). The initial receptive fields were recovered. (*f*) Fraction of cells selective to the same feature before and after perturbation for different degrees of perturbation. In all ensembles with at least two co-tuned cells, feed-forward inputs were scrambled for a variable fraction of the ensemble neurons: blue bars: randomized feed-forward inputs of a certain percentage of ensemble cells; white bar: all feed-forward and all recurrent weights were set to their random initial values. (Mean: average over 100 trials; error bars: standard deviation of the mean.)

To test if such a network structure could convey resistance to drift and perturbations, we used a previously described spiking network model implementing a biologically plausible voltage-dependent spike-timing-dependent plasticity rule [91]. In contrast with other models using different plasticity mechanisms, this model closely reproduces the signature structure of sparse connectivity with few strong reciprocal connections between co-tuned neurons [91]. As described in Clopath *et al.* [91] and figure 3, the excitatory neurons in the model network develop selective stimulus preferences and cells with similar feature selectivity form subnetworks by developing reciprocal connections (figure 3*b*).

To assess the long-term stability of single-cell feature selectivity in this artificial network, we allowed the model to continue to run for a further 10 000 iterations. Even though synapses fluctuated between strong and weak weights [91], the overall connectivity remained stable on average and, importantly, receptive fields showed only minimal drift (figure 3*c*, cf. figure 1*b*).

To test the perturbation resistance of the model network, we identified individual interconnected subnetworks (i.e. ensembles) of two or more cells that were selective for the same input feature. We then simulated perturbations of individual ensembles by reinitializing the feed-forward input weights of a variable fraction of the respective ensemble neurons with random values (figure 3*d*). We left the recurrent weights unchanged—but still plastic—and then let the network re-converge to a new stable state (figure 3*e*). We found that we could scramble the feed-forward inputs of as many as 75% of all cells in an ensemble and still largely recover the initial feature selectivity and recurrent connectivity matrix of the subnetwork (figure 3*f*). We therefore conclude that in this model a ‘backbone’ of strong recurrent connectivity is able to guide the recovery of initial feature selectivity even when a large fraction of the feed-forward weights have been randomly changed. Robust recovery, however, only occurs if a small fraction of cells in an ensemble maintains its original selectivity (figure 3*f*). Such non-plastic ‘anchor’ cells effectively propagate the initial ensemble selectivity to the remaining interconnected neurons. Indeed, we recently found that only two-thirds of excitatory L2/3 neurons underwent robust plasticity whereas the others did not change their eye-specific tuning [15]. It is tempting to hypothesize that some of these cells would act as ‘anchors’ for their respective ensembles to guide precise recovery from perturbations [15]. Without further experimental evidence, of course, such an interpretation remains speculative. From the perspective of computational modelling, it is also conceivable that perturbation resistance could be achieved by initially stable feed-forward weights together with scrambled recurrent connectivity. At least in the case of OD plasticity, however, especially the feed-forward weights in the shape of thalamocortical input to L4 of visual cortex have been shown to be sites of prominent plasticity after MD [92], rendering this alternative biologically less plausible.

## 7. Summary and outlook

In recent years, several studies chronically followed the activity of individual neurons employing a variety of experimental paradigms in different brain regions. Most of these studies agree that neuronal representations are stabilized with increasing behavioural relevance of the respective stimuli during learning. Recent data also provide an ever more fine-grained picture of neuronal long-term stability, showing that representational stability is differentially regulated even in nearby circuits [67], in excitatory and inhibitory neurons [37,55], in different projection neuron types [39] and in cortical sublayers [40,48].

There is less agreement, however, regarding the degree of absolute variability of neuronal representations and the magnitude of long-term drift, i.e. the degree to which population activity becomes progressively more dissimilar over time (figure 1). In general, chronic Ca^2+^ imaging studies report a larger level of variability and drift than longitudinal electrophysiology (cf. [22,27]). Potential reasons for this are different biases towards highly active and less active neurons, linear and nonlinear activity readouts, and ambiguous and non-ambiguous long-term identification of cells for electrophysiology and imaging, respectively (for further discussion see [16,22,34]). However, these explanations will remain largely speculative for as long as these indispensable contemporary techniques are not compared directly and ideally simultaneously. It would be highly desirable to chronically record from the same neuronal population using implanted electrodes together with different chronic Ca^2+^ imaging techniques. While certainly challenging, a recent study demonstrated that this should be technically feasible [93]. Without further quantification of the measurement idiosyncrasies of different recording techniques, relative measures of variability (e.g. of drift) remain preferable to measures of absolute variability.

A further unexplored question is to which degree representational variability is different across species, as, for instance, has already been suggested for the hippocampus [69,75,76]. Especially in the sensory cortex of animals that show a more defined ‘critical period’ for plasticity early in life, e.g. cats or monkeys [79,80], variability in mature circuits may be lower than in mice. Clearly, it would be very important to perform comparative chronic recording experiments in various animal models.

A further unanswered question concerns the role of structural and functional plasticity for neuronal stability. Is plasticity during normal sensory experience rather stabilizing or destabilizing? Is, for instance, baseline spine turnover a sign of circuits constantly integrating error signals to readjust to the sensory statistics of the outside world, thereby maintaining stability owing to constant feedback [62]? Or is plasticity rather destabilizing, with a certain level of unavoidable constitutive plasticity ‘noise’ that may lead to drift if not compensated for by, e.g. specific network structures (figure 3)? These questions could be partially addressed by correlating structural changes with functional changes on a single neuron level or by boosting or suppressing plasticity on the population level while chronically recording the stability of single-cell representations. Recent data suggest that, indeed, the level of synaptic turnover correlates with the level of representational drift [9]. In accordance with a large degree of long-term drift [28,52,66], most dendritic spines in hippocampal CA1 have a lifetime of only a few days [9] whereas a large fraction of spines in the neocortex are long-term stable [8,10,94,95].

At least in visual cortex, the overall level of activity and the tuning of individual neurons are surprisingly invariant, with both features resisting even strong circuit plasticity after reversible chronic modification of sensory input [15,23]. We provide a speculative circuit model that shows that stable baseline representations and precise recovery from perturbations could be achieved with a ‘backbone’ of strong recurrent connectivity between similarly tuned cells together with a small number of non-plastic ‘anchor’ neurons (figure 3). Although measures of similarity of neuronal correlation structures provide indirect evidence for maintenance of the initial network structure after recovery from plasticity [15], more refined techniques would be necessary to probe the degree of recovery of individual interconnected ensembles, as we could do in the model (figure 3*f*). Recently emerging all-optical techniques may provide this opportunity in the future. Patterned two-photon photostimulation of light-gated cation channels together with simultaneous Ca^2+^ imaging of activity could potentially be used to both chronically map the stimulus selectivity of individual neurons and also their effective functional connectivity during baseline and experience-dependent plasticity [96,97].

Together with the large-scale efforts to map cellular activity chronically with regional and cell-type specificity [98] and constant improvements of our toolset to label and record from cell types defined by their genetic makeup [99] and their specific set of synaptic input and output patterns [100], we are confident that the coming years will dramatically enhance our knowledge of the role of variable and stable neuronal representations for cortical function.
